# Understanding Global Rice Trade Flows: Network Evolution and Implications

**DOI:** 10.3390/foods12173298

**Published:** 2023-09-02

**Authors:** Wei Chen, Xiquan Zhao

**Affiliations:** 1 Institute of Geographic Sciences and Natural Resources Research, Chinese Academy of Sciences, Beijing 100101, China; 2 College of Resource and Environment, University of Chinese Academy of Sciences, Beijing 100049, China

**Keywords:** rice trade, food trade, trade network, food supply chain, food security

## Abstract

Rice holds a significant position as one of the world’s most important food crops, and international trade plays a crucial role in regulating rice supply and demand. Analyzing the structural evolution of the global rice trade from a network perspective is paramount for understanding the global rice-trade supply chain and ensuring global food security. This study utilizes international rice-trade data from 2000 to 2021 and employs various network analysis methods to depict the spatial and temporal patterns of the global rice trade, examines the network topologies of the global rice trade, and reveals the impacts of its evolution on food security. The research findings are as follows: (1) Global rice-trade scale has increased over time, indicating a relatively stable development with the gradual formation of complex rice-trade networks. Since 2000, the global rice-trade networks have shown increasing density characterized by Asia as the primary export source and Africa as an important import market. (2) Network analysis indicators demonstrate a growing trend in the size and density of the global rice-trade networks, along with increasingly optimized network structures and improved network connectivity efficiency. Core positions in the networks are occupied by Thailand, Vietnam, India, China, Pakistan, and the United States, while import partners in European and American countries, such as Germany, France, UK, Canada, The Netherlands, and Belgium, show greater diversification. Asia, Europe, and North America form agglomeration regions for rice-exporting countries. Additionally, importing and exporting countries in the global rice-trade networks exhibit certain geographical concentrations. (3) The network backbones of the global rice trade are continuously evolving and being refined, characterized by dominant large rice-exporting countries in Asia and prominent developed countries in Europe and North America. The backbone structures revolve around India as the core, Thailand and Pakistan as the second cores, and critical nodes represented by Italy, the United States, China, and Vietnam. Regional backbone networks have also formed in Asia and Europe. Based on these findings, this paper clarifies the complex network characteristics of the global rice trade and offers insights to promote international rice-trade cooperation and safeguard global food security.

## 1. Introduction

Food is the material basis for human survival and a vital strategic resource for maintaining social stability and promoting economic development. Food security has become an important part of national security and has received extensive attention from decision-making authorities and academics worldwide. Influenced by resource endowment, natural conditions, production technology, cultural habits, and other factors, global food production and consumption of various types of food are characterized by significant geographical roots, making foreign trade a critical means of regulating the relationship between food supply and demand [1]. According to the Food and Agriculture Organization of the United Nations (FAO) statistics, in the past three decades, the global food-trade volume has more than doubled, the inter-country food-trade links are becoming increasingly close, the food security of each country and region are associated with international food trade to varying degrees, forming a complex global food supply-chain system. However, global food trade and the security of supply are facing increasingly serious challenges. The rapid growth of the world’s population has led to a further increase in food demand [2]; climate change has exacerbated the frequency and intensity of extreme weather events, posing a threat to food production [3]; unstable events such as the COVID-19 outbreak and the Russia–Ukraine conflict have been frequent, and export restrictions have been implemented in some economies, while wheat and other food prices have risen, exposing some countries and regions to the risk of disruption in food supply [4,5]. Among them, rice, as one of the three major staple grains, faces the same challenges in its production, supply and consumption, and the stable supply of rice globally plays a key role in maintaining world food security. In this context, it is of great significance to clarify the global rice trade’s development trends, understand global rice-trade flows and ensure the supply chain’s stability to guarantee global food security.

Rice is one of the most important food crops in the world and a raw material for brewing and sugar production. In 2021, the production and consumption of rice accounted for about 18.6 and 18.7% of the world’s three major food crops, and the consumption of rice accounted for about 81.3% [6]. It has become the main food source for over half of the world’s population. It provides some of the poorer populations in Asia, Latin America, and Africa with up to 50% of their dietary calories and a significant portion of protein intake for some poorer populations in Asia, Latin America and Africa [7], playing an important role in maintaining social stability and alleviating hunger. The production and consumption of rice have a certain spatial imbalance, with East, Southeast and South Asian countries such as China, India, Indonesia and Vietnam being the major producers of rice, with China and India alone accounting for about half of the rice cultivation [7], while rice-consuming countries are relatively decentralized and globally dispersed, making international trade an important way to regulate the supply and demand for rice. The existing literature related to the rice trade mostly focuses on specific countries and regions, and the main research topics include the international trade patterns in rice and the characteristics of partner countries [8,9], the impacts of elements such as trade agreements and extreme weather on the rice trade [10,11,12], and the flow of virtual water and the depletion of land resources in the rice trade [13,14], etc. The relevant research provides a reference for optimizing international rice-trade cooperation and enhancing food-supply security.

As a result of the separation of rice production and consumption, international trade in rice has become the primary way to maintain the balance of food supply and demand in all countries of the world, and international rice-trade links have been continuously strengthened, gradually forming a complex and nested global rice trade network system. Therefore, how to accurately identify the changes in global rice production and consumption patterns from the network perspective is not only conducive to comprehensively revealing the evolution laws of the global rice-trade flows, but also an objective need to safeguard global food security and the stability of rice supply under the complex international situation. Accompanied by the rise in network science, the complex network analysis method can quantitatively assess the trade links between countries [15,16], which provides cutting-edge analytical approaches for exploring global-trade patterns and development trends. In this context, academics have gradually begun to draw on complex network analysis to analyze the patterns and evolution of the global food trade from a network perspective, with research topics focusing on the pattern evolution of the food-trade network [17,18], the differences in the trade patterns of different crops [19], and the vulnerability and risk in food trade and supply [20,21,22]. Among them, the rice trade, as an essential component of the food-trade system, has also received some preliminary exploration. Zhang and Zhou used network analysis to explore the micro features of the major global food-trade networks and found that more and more economies are involved in the global rice trade, with countries with high rice production, such as India, having greater control over rice exports, and developed economies, such as Germany, The Netherlands and Italy, occupying a prominent position in rice imports [19,23]. Burkholz and Schweitzer constructed global-trade networks of four food crops, such as rice, to simulate the response of the network under different shock scenarios and found that some Asian countries have prominent positions in the global rice-trade network. India and Thailand are decisive for rice supply in regions such as Africa, while trade shocks from the United States are closely related to Asian countries such as Japan and South Korea [24]. As research in this area progresses, scholars have expanded their analysis beyond the global food-trade network. Some have delved into examining changes in international and regional rice-trade patterns and network structures from the perspective of specific countries, such as China. Preliminary conclusions have been drawn, shedding light on the implications of maintaining a balance between rice supply and demand [25,26]. However, it is noteworthy that the existing literature predominantly focuses on the food-trade network, including the rice trade, without giving adequate attention to comprehensive and systematic studies exclusively dedicated to the global rice-trade network. Consequently, the overall impacts of the evolution of the global rice-trade network on food security have not been thoroughly assessed. Complex interactions distinguish the global rice-trade network, and its structure is undergoing dynamic evolution due to the influence of climate change and the complex and volatile international situation.

The importance of understanding the dynamics and complexities of the global rice-trade network becomes evident in the context of maintaining global food security. Therefore, to address this research gap, this study constructs global rice-trade networks based on the UN Comtrade Database, comprehensively employs various network analysis methods in order to depict the spatiotemporal evolution of the global rice trade and analyze the topological structure of the global rice-trade network. The study aims to clarify global rice-trade flow patterns from a network perspective and offer valuable insights for promoting the international rice trade and maintaining global food-supply security.

## 2. Methods and Data

### 2.1. Analytical Framework

#### 2.1.1. Network Density

Network density is one of the most commonly used network attribute measures to gauge the closeness of network connections. Network density can be expressed as the ratio of the number of edges present in the network to the maximum possible number of edges, and the higher the network density, the more tightly connected the network is. For undirected networks, the formula for network density is [27]:

(1) $$D=\frac{2M}{N(N-1)}$$

where *M* is the number of edges present in the network, and *N* is the number of nodes.

#### 2.1.2. Global Clustering Coefficient

Global clustering coefficient is a parameter that measures the degree of clustering of network nodes. The clustering coefficient of a single node is defined as the ratio of the number of edges between all its neighboring nodes to the maximum number of possible edges [28]. The formula is:

(2) $$E_{i}=\sum_{j,k,h\in n_{i}}e_{jk}e_{kh}$$

(3) $$C_{i}=\frac{2E_{i}}{k_{i}(k_{i}-1)}$$

where $n_{i}$ is the set of neighboring nodes of node *i*, $E_{i}$ denotes the actual number of edges between neighboring nodes, $k_{i}$ is the number of edges of node *i*, and $C_{i}$ is the clustering coefficient of node *i*. A larger value $C_{i}$ indicates that the neighboring nodes of node *i* are more interconnected.

Global clustering coefficient of the network C is the average of the clustering coefficients of all the nodes, which is calculated as follows:

(4) $$C=\frac{1}{N}\sum_{i=1}^{N}C_{i}$$

The larger C is, the greater the degree of agglomeration between the nodes in the network and the greater the degree of forming short-distance links between the nodes.

#### 2.1.3. Global Efficiency

The efficiency between any two nodes *i* and *j* in a network is defined as the inverse of the shortest path between nodes *i* and *j*. Global efficiency, which represents the network’s overall transmission capacity, is the average efficiency across all pairs of nodes. The shorter the shortest paths between nodes in a network, the greater the global efficiency is, and the faster the transfer rate between nodes. The global efficiency is calculated as [29].

(5) $$E_{g}=\frac{1}{N(N-1)}\sum_{i\neq j}\frac{1}{d_{ij}}$$

where N is the number of nodes in the network and $d_{ij}$ the distance between any nodes *i* and *j*.

#### 2.1.4. Degree Centrality

Degree centrality is often defined as a simple count of the total number of connections linked to a vertex. Degree centrality is an index that portrays the strength of a particular node’s connection with other nodes. The greater the degree centrality, the stronger the node’s connection with other nodes and its position in the network. In weighted networks, degree centrality is usually expressed in terms of the strength of a node, also known as weighted vertex degree (WVD), which is computed as [30]:

(6) $$WDC_{i}=\sum_{j\in n_{i}}W_{ij}$$

where $W_{ij}$ denotes the weight of the edges between node *i* and other node *j* in the network.

In directed networks, degree centrality is divided into in-degree and out-degree. The following relationship exists between out-degree, in-degree and degree centrality:

(7) $$DC_{i}=DC_{i}^{in}+DC_{i}^{out}$$

In the global rice-trade network, a country’s weighted degree centrality indicates the country’s total foreign trade in rice, with the in-degree used to measure the number of the country’s rice importing partners and the out-degree indicating the number of rice exporting partners.

#### 2.1.5. Disparity Filter

In a network, the backbone structure is a sparse, (un)weighted subgraph containing only the most “important” or “significant” edges of the network. Extracting the backbone of a network is very effective in understanding the network’s structure when the original network is too dense, or the edge weights are difficult to interpret. In particular, the disparity filter algorithm [31] exploits local heterogeneity and local correlation to filter out the dominant connected backbone structure in a weighted network with strong disorder and retains the structural properties and hierarchies at all scales. As a result, the disparity-filter algorithm greatly reduces the number of edges in the original network while retaining almost all of the weights and a large fraction of the nodes [32].

To evaluate the effects of weights in a network that are not balanced on a local scale, for each node i with k connected nodes, a computational function can be derived:

(8) $$\omega_{i}\left(k\right)=kY_{i}\left(k\right)=k\underset{j}{\sum }p_{ij}^{2}$$

where $Y_{i}\left(k\right)$ indicates the degree of local heterogeneity. In the case of complete homogeneity, the $\omega_{i}\left(k\right)$ is equal to 1, while in the case of complete heterogeneity, the function is $\omega_{i}\left(k\right)$ = k.

The null model is often used to define abnormal fluctuations and provides the expected value of a measure of variation at a given node in a purely random situation. For the variable value x, the probability density function is:

(9) $$\rho \left(x\right)dx=\left(k-1\right){\left(1-x\right)}^{k-2}dx$$

depends on the node degree k under consideration.

The disparity filter algorithm extracts the backbone structures by identifying which node connections should be preserved. Statistically relevant edges will be those whose weights satisfy the following relationship:

(10) $$\alpha_{ij}=1-\left(k-1\right)\int_{0}^{p_{ij}}{\left(1-x\right)}^{k-2}dx<\alpha$$

note that this expression depends on the number k connections of the nodes connected by the considered edges.

### 2.2. Data Source

This paper takes the rice trade as the research object and adopts the bilateral trade flow data between countries and regions from the UN Comtrade Database, and the HS code for “rice” trade data is 1006, including the information on importing countries, exporting countries, trade value and trade volume. Rice has both natural and commodity properties, and its price is significantly affected by trade policies, climate change, pandemics, financial crises and regional conflicts and fluctuates greatly over time [33]. Therefore, to avoid the interference of rice-price changes in different years on the research results, this paper considers the time series changes in rice-trade value and trade volume at the same time, and mainly adopts the trade volume to explore the evolution characteristics of the spatial patterns and topological structures of the global rice-trade networks. Finally, through the cleaning and transformation of import and export data, this paper constructs the global rice-trade network dataset since 2000, covering 216 countries and regions and 46,656 values of trade relations worldwide, and selects 2000, 2010, 2015 and 2021 as representative years to carry out the whole study. In this study, we construct initial directed and weighted networks with countries and regions as nodes, inter-country trade links as edges, and the inter-country trade volume as weights, and apply different forms of the networks to conduct the research, respectively.

## 3. Results and Analysis

### 3.1. Temporal Changes in the Global Rice-Trade Scale

**Figure 1:**
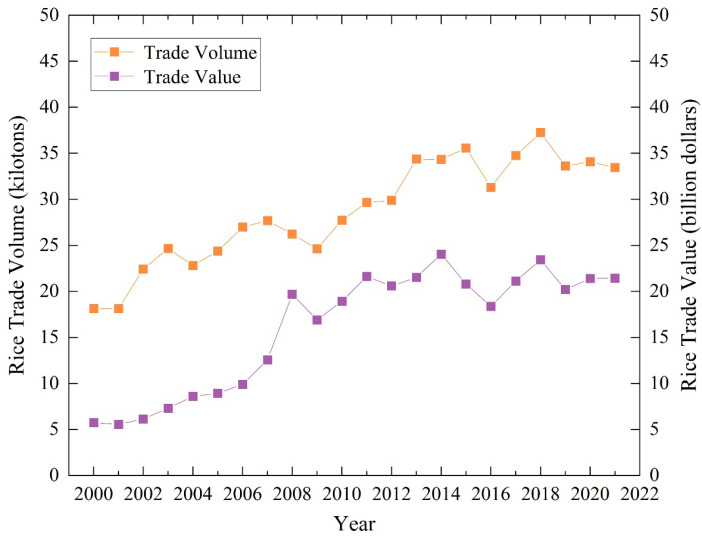
Temporal changes in the global rice trade volume and value.

Figure 1 presents the time-series evolution of global rice-trade volume and trade value from 2000 to 2021. It shows an overall fluctuating growth trend for both trade volume and value. From 2000 to 2007, the trade value of rice increased annually, while the trade volume fluctuated, growing from USD 5.76 billion and 18.13 kilotons to USD 12.56 billion and 27.69 kilotons, respectively. China’s accession to the WTO in 2001, as a significant rice-growing and consuming country, contributed to the expansion of the global rice trade due to trade liberalization [34]. However, in 2008, droughts and extreme weather conditions reduced rice production, while the financial crisis and increased food consumption in the industrial sector resulted in a sharp increase in global rice prices. This, in turn, led to rapid growth in the global rice trade in 2008, followed by a contraction in 2009. From 2009 to 2021, global rice-trade volume and trade-value trends became more consistent with increased volatility. After the food crisis, rice prices declined, and the global rice market regained stability. In 2015, sufficient rice supply, reduced fertilizer and transportation costs, and lower crude oil prices contributed to decreased global rice prices and increased trade volume [35]. However, in 2016, severe droughts in South Asia and Southeast Asia, particularly in large rice-producing countries like India and Vietnam, led to reduced production, further affecting the size of the global rice trade. Trade frictions between countries caused a decline in the scale of the global rice trade in 2019. During the COVID-19 pandemic, some countries implemented export restrictions and shipment suspensions, increasing international rice prices. In addition, USDA statistics show that since 2020, higher prices for seeds, fertilizers, and fuel have significantly increased the cost of rice production borne by major producers, squeezing producers’ profits and reducing incentives for producers and rice farmers to plant, posing a potential threat to the stable supply of rice and food security. Nevertheless, the overall development of the global rice trade remained relatively stable, highlighting the importance of a smooth and stable food trade and supply chain for socio-economic recovery in the post-pandemic period.

### 3.2. Spatial and Temporal Evolution of the Global Rice Trade

**Figure 2:**
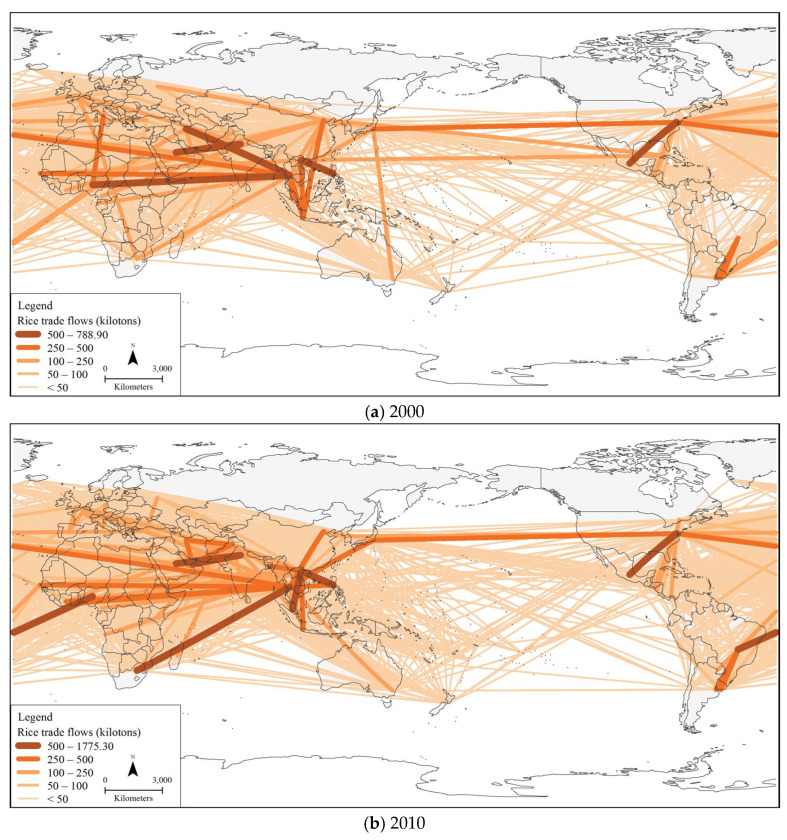

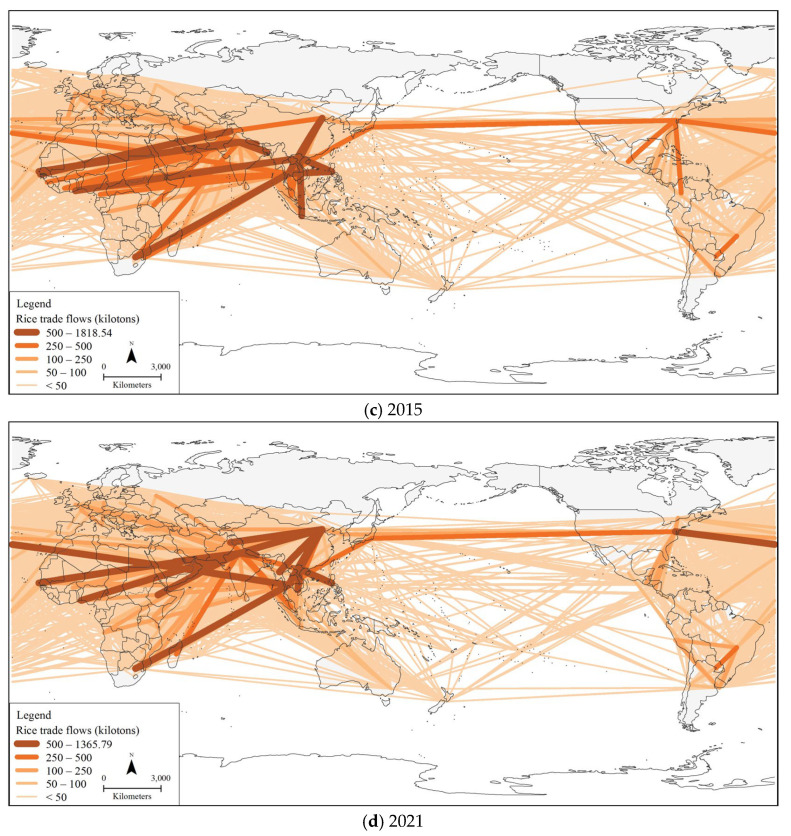
The spatial and temporal evolution of the global rice trade.

This paper takes countries as nodes, rice-trade links between countries as edges, and bilateral trade volume as the weights of edges, and constructs the global rice-trade networks for the four years of 2000, 2010, 2015, and 2021. The results are shown in Figure 2. Overall, the global rice-trade networks have been in dynamic evolution since 2000, and the network size and density have continued to increase, gradually forming network patterns with clear hierarchical features and obvious spatial imbalances.

In terms of overall characteristics, the global rice-trade networks have undergone hierarchical expansion, with the hierarchical structures and agglomeration characteristics of trade flows becoming more pronounced. Rice trade has an increasingly significant polarization effect, with a small number of trade links occupying most of the trade volume. Between 2000 and 2021, the number of links in the network with a size larger than 50 kilotons accounted for less than 5% of the total number of links at all times, while the trade volume accounted for 82.24% of the total trade volume, up from 74.01%. The global trade in rice has a prominent differentiation phenomenon, and the degree of differentiation has been elevated over time. The scale of trade links at each network level has increased, resulting in an increasing trend in the number of links at each level. From 2000 to 2015, the number of links with trade sizes of more than 500 kilotons, 250–500 kilotons, 100–250 kilotons, 50–100 kilotons, and less than 50 kilotons increased from 5, 10, 27, 32, and 1706 links to 13, 21, 43, 57, and 2720 links, respectively. And the scale of trade between countries that have already cooperated in the rice trade has generally increased, with more and more countries establishing new trade linkages. As a result of trade frictions and the COVID-19 outbreak, the scale of the rice trade between some countries has shrunk in recent years, with the total number of links larger than 250 kilotons decreasing to 23 in 2021, and there has been a marked decline in the scale of rice trade between some Asian countries and African countries.

Regarding spatial linkages, the global rice-trade networks are undergoing dynamic evolution, with increasing inter-country trade links and Africa and Asia becoming the concentration areas for the rice trade. Since 2000, a rice-trade network pattern has emerged with Asia as the primary source of exports, Africa as the main market for imports, and a general increase in the participation of the remaining regions. Asian countries such as India, Thailand, Vietnam, Indonesia, China and Pakistan are major rice exporters, while African countries such as Ethiopia, Benin and Senegal are major importers. Countries in sub-Saharan Africa generally rely on rice as a staple food, and rice is one of the major crops there. Still, frequent droughts, volatile situations, low rice production and processing levels, and population growth have resulted in insufficient locally produced rice to meet consumption demand [36]. The annual growth rate in rice consumption is much higher than that in production, which has led to the search for rice imports [37]. Compared with 2015, the scale of the rice trade between some African and Asian countries in 2021 has shrunk, making some African countries face a more serious hunger problem. The reason for this is, on the one hand, that the COVID-19 pandemic has had a severe impact on the economy of sub-Saharan African countries such as Nigeria, leading to a decrease in the foreign exchange reserves used by the countries to purchase rice [36], and, on the other hand, countries such as Vietnam, Myanmar, and India, in order to ensure domestic supply considerations during the pandemic period, took measures to restrict the export of rice, reducing the volume of rice exports to African countries. Mutual trade between countries in South, Southeast, and East Asia also plays an important role in the global rice-trade network. Differences in the levels of rice production and demand among Asian countries are an objective need for rice-trade cooperation, while the signing of regional trade agreements such as the ASEAN Free Trade Area (AFTA) and the South Asian Free Trade Area (SAFTA), as well as the establishment of international cooperation platforms such as the Belt and Road Initiative (BRI), have facilitated the bilateral trade in rice among Asian countries. Among the major rice-importing countries in Asia, Nepal lacks high-quality seeds and fertilizers as well as sound irrigation facilities, making it difficult for domestic rice production to be self-sufficient [38]; countries such as Singapore are less likely to grow rice due to the limitations of its industrial structure and land area; and countries such as China and the Philippines, which are large rice-producing countries with large populations, import rice from neighboring countries to ensure the security of food supply [25]. The United States is a major North American rice-trading country, and its trade partners have changed considerably over time. Among them, the size of the bilateral rice trade between the United States and Thailand has nearly tripled from 276.59 kilotons in 2000 to 543.96 kilotons in 2021, while the size of the rice trade between the United States and Mexico has decreased significantly. In the framework of the North America Free Trade Agreement (NAFTA), rice from the United States can enter the Mexican market duty-free, making the United States Mexico’s largest source of rice imports, but with the increasingly lower prices of rice from South American countries such as Brazil and Uruguay, Mexico’s leading source of rice imports is gradually shifting to the South American countries. In addition, some countries in Oceania and Latin America have always been at the relative periphery of the global rice-trade networks, with crops such as wheat and maize being the main foodstuffs in these regions, and rice production and consumption being relatively small.

### 3.3. Network Topologies of the Global Rice Trade

Based on the network indicators of graph size, network density, global clustering coefficient and global efficiency, this paper portrays the macroscopic characteristics of the global rice-trade networks and their evolution trend (Table 1). The results show that: (1) The graph sizes of the global rice-trade networks show a steady growth trend, expanding about 1.6 times since 2000, indicating that the inter-country trade links have gradually increased, and the rice-trade partners of some countries are becoming more diversified. (2) The network density is increasing from 0.0767 to 0.1242, year by year, indicating that the global rice-trade networks exhibit a densification trend, and the trade links between countries are becoming increasingly close. (3) The global clustering coefficient experienced stable growth first and then an increase in the growth rate. From 2000 to 2015, the global clustering coefficient grew steadily from 0.3583 to 0.3923; the agglomeration of the global rice-trade network deepened, and the tendency for trade links with the two major regions of Asia and Africa became evident. From 2015 to 2021, the global clustering coefficient grew sharply to 0.4433, with China, Vietnam, India, Myanmar, and other countries as the core of the maturing Asian rice-trade group. Trade links within the group are becoming increasingly close. The downsizing of the scale of rice imports into African countries has made the trend of the global rice-trade networks to Asia and other regions even more apparent. (4) The global efficiency shows fluctuating growth followed by rapid improvement, indicating that the expansion of the network scale has made the connectivity, accessibility and transmission efficiency of the network increase accordingly. It is worth noting that, between 2015 and 2021, the global efficiency of the network was significantly improved while the graph size increased only slightly, indicating that establishing trade links between key nodes effectively impacts the improvement of network connectivity. Overall, the global rice-trade networks are becoming increasingly complex and dense, while the network structure is increasingly optimized and the network efficiency is significantly improved.

To measure the positions of countries in the global rice-trade networks, we extracted the top 10 countries and their values of weighted degree centrality (WDC), in-degree (IN_D), and out-degree (OUT_D) in the years 2000, 2010, 2015, and 2021 (Table 2). The indicators show that the main importing and exporting countries of global rice trade have a certain geographical concentration, and some Asian, African, European and American countries play essential roles in the trade networks.

With the help of weighted degree centrality, we can identify the core nodes in the networks with high-trade scale and large influence. Since 2000, Thailand, Vietnam, India, China, Pakistan and the United States have occupied the core positions in the global rice-trade networks, and the trade scale of rice has always been at the forefront. India, Thailand, Vietnam and Pakistan have many export-partner countries and have become large rice producers and exporters due to their good soil and water resources. The size of China’s trade declined between 2000 and 2010 and has since grown significantly as rice imports have risen. China’s import trade is larger than its export trade due to the strong demand and tight supply and demand in its domestic rice market [25]. Still, China’s rice import sources are concentrated in a few countries, with the number of importing partner countries being only 16 in 2021, while the number of exporting partners is as high as 117. China is one of the major rice markets for neighboring countries such as South Korea and Japan and African countries such as Côte d’Ivoire, as well as for large rice-producing countries such as Vietnam. The US has a central position in the global rice export and import systems, with more than half of the world’s export-partner countries involved, and the US has become the core of the high-quality rice export market through its advanced cultivation and management technologies. In all years, West African countries were ranked among the high-value countries in weighted degree centrality, but the core countries in West Africa’s rice trade are in dynamic change. In 2000, Nigeria was the largest country in Africa’s rice trade. To reduce its dependence on rice imports, Nigeria implemented an import substitution policy, and the Buhari government proposed the “Anchor Borrowers’ Program”, which provided farmers with quality rice seeds and start-up capital to grow rice [39], aimed at increasing domestic rice production, which led to a reduction in the size of rice imports year by year. In 2015, Senegal and Côte d’Ivoire succeeded Nigeria as the core of the rice trade in Africa. The high rice import dependence of the two countries has caused the governments and people to think about and pay attention to food security, thus taking measures to increase domestic rice production and moderately reduce the scale of rice imports. In Senegal, the government has invested in fertilizers and agricultural infrastructure to improve rice self-sufficiency and quality, which has boosted local rice production to some extent, while Côte d’Ivoire has improved its rice self-sufficiency through the introduction of new varieties and upgraded cultivation techniques. In 2021, the core of Africa’s rice trade is shifting to countries such as Ethiopia and Benin.

The in-degree of a node can reflect a country’s position and role in the global rice-import system. Countries with a high number of rice-import partners are concentrated in Europe and North America, including Germany, France, the UK, the United States and Canada. Over time, European countries such as The Netherlands and Belgium have increased their positions in the global rice-import system, while South Africa’s position has declined slightly. Europe is not a major rice-producing and consuming area compared to Asia and Africa. Although countries such as Germany, France and the UK have a relatively large number of rice-importing partner countries, the scale of their imports is not prominent globally. In addition to being utilized for animal feed and industrial production, imported rice is mainly used to satisfy the dietary demands of small-scale groups such as ethnic minorities. The Netherlands and Belgium are trade gateways to Europe, with world-class ports such as the Ports of Rotterdam and Antwerp, which are hubs for rice shipments into Northwest Europe from Asian countries such as Thailand, India, and Vietnam. At the same time, The Netherlands and Belgium also rank among the top European countries in terms of growth in rice consumption, and residents in both countries have a positive attitude towards rice consumption, believing that rice is healthy, cheap, easy to digest and a good alternative to staple foods such as potatoes. South Africa has always been one of the African countries with the largest number of rice-import partner countries, stabilized at about 43 since 2010. In 2021, the COVID-19 pandemic caused some Southeast Asian countries to reduce rice production and impose export restrictions affecting the stability of the global rice supply. Some of the major importing countries enhance the stability of the rice supply by increasing the number of import partner countries, expanding the import trade channels and other measures, so that the countries of the in-degrees of nodes increase. However, the number of import-partner countries of South Africa has not significantly increased, resulting in a decline in its relative position in the global rice-trade networks.

The node out-degree can be used to locate the trade network’s primary export nodes. The main exporting countries in the global rice-trade networks are concentrated in Asia, Europe and North America, which is consistent with the global rice production pattern, and the position of each country in the rice export system is generally more stable. As rice cultivation is subject to greater constraints in terms of thermal and water conditions, the global rice production areas are more concentrated, resulting in the global rice export pattern rather than the import pattern having a more significant polarization effect and spatial agglomeration characteristics; the node out-degrees of the core exporting countries are much larger than the node in-degrees of the main importing countries. A small number of large exporting countries have strong control over the spatial pattern of the global rice-trade network and the overall direction of the rice flow. Specifically, in addition to the core countries with large trade sizes, such as India, the United States, Thailand, China, Vietnam, and Pakistan, countries such as Italy, Spain, France, and Japan also have many export-partner countries. Rice cultivation in Europe is limited to a few southern European countries. Italy and Spain are two of the largest rice-growing countries in Europe, accounting for about 75% of Europe’s rice production. Of these two countries, Italy’s rice exports accounted for more than two-thirds of the whole of Europe; the main export markets are concentrated on the Mediterranean coast and Eastern Europe and other neighboring regions, but other regions of Europe are an important source of high-quality rice imports. Compared with other Asian countries such as China, India and Vietnam, Japan’s rice planting area is small, but the level of yields has steadily increased, due to leading planting technology and mechanization levels, targeting the high-end market, and thus becoming one of the main exporters of high-quality rice.

### 3.4. Network Backbones of the Global Rice Trade

**Figure 3:**
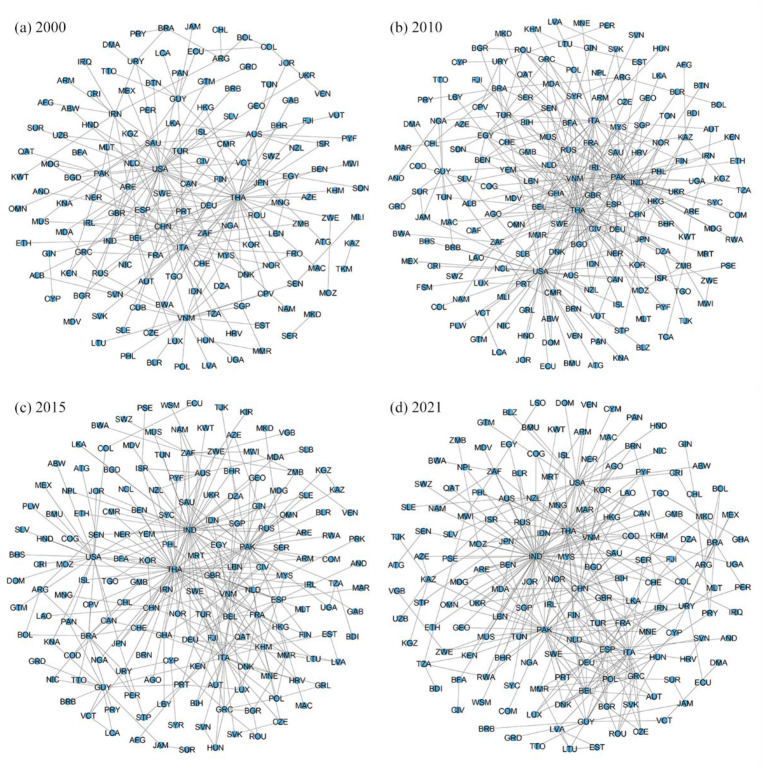
The network backbones of the global rice trade.

In the global rice-trade networks, there are many small-scale trade links, which have no significant impact on the overall function and normal operation of the networks, but may obscure the network’s key features and main structure. In this paper, the disparity filtering algorithm is applied to eliminate the low-influence nodes and edges to avoid their interference with the network features while preserving the main structure, key features and main information of the network, identifying the backbone structure that supports the operation of the network (Figure 3).

In terms of backbone edges, the number of backbone links in the global rice-trade networks has been increasing, and the backbone structure has shown a trend of densification, with the backbone network within Asia being more stable and the European rice-trade backbone networks gradually coming to the fore. In 2000, 238 links were identified as backbone links, accounting for only 13.36% of the original rice-trade network but accounting for as much as 87.96% of the trade size. Over time, the backbone structures of the global rice-trade networks have further expanded and enriched, with 331, 330 and 345 backbone edges in 2010, 2015 and 2021, respectively. Among them, trade links with a size larger than 50 kilotons in the original network have only ten or fewer not identified as backbone links each year, while less than 10% of trade links with a size below 50 kilotons are retained in the backbone structures, indicating that larger trade flows play a major role in supporting the main structures of the rice-trade networks, while smaller trade flows mostly play a limited role in the network; some of the smaller trade flows are also classified as dominant edges because they bear most of the rice-trade size of the marginal countries in the network. In terms of the trade scale, the bilateral rice-trade scale between countries in East, Southeast and South Asia has always been at the top since 2000, and a stable localized backbone network has been formed within Asia, with countries such as India, Thailand, Vietnam, China, and Pakistan at the core. The scale of rice exported from Asian countries to West Africa has also increased since 2015, and most of the related trade links have been identified as backbone links. The trade links between the two regions of Asia and Africa occupy a certain position in the backbone structure. However, African countries have a relatively small number of backbones due to having a single rice-importing partner, being relatively peripheral to the backbone networks, and not forming a clear trade bloc. In contrast, the scale of the rice trade among European countries is not prominent in the original network, but there are more trade links identified as backbones, forming a backbone structure around countries such as Italy, Spain, Poland, Germany, Greece and Belgium, with a developing European rice-trade bloc, and a European rice-trade backbone network that has grown progressively tighter and more prominent over time.

From the viewpoint of backbone nodes, the backbone structures of the global rice-trade networks show a tendency to concentrate in a few countries and regions, and the core nodes of the backbone structures change slightly over time, with India’s core position gradually coming to the fore, the positions of the US, Thailand, and China adjusted, and countries such as Pakistan, Italy, and Vietnam always remaining important nodes in the backbone networks. In 2000, the backbone edges of Thailand and the US were 36 and 34, respectively, and the two cores radiating to drive other regions worldwide. After 2010, the positions of Thailand and the US in the backbone network slightly declined. Thailand’s backbone connectivity decreased from 58, in 2010, to 35, in 2021, dropping to second place and losing its core position. The reason for Thailand’s decline was that the rice pledging policy implemented during Yingluck’s administration did not achieve the expected goal of raising global rice prices, but rather caused Thailand’s rice to lose its price advantage in the international market. Its export market has been taken over by neighboring rice-exporting countries such as India, Vietnam, and Pakistan. The number of US backbone links was reduced to 22. Its trade links with Latin American and African countries such as Nicaragua, Jamaica, Ecuador, and Ghana were no longer recognized as backbone links. The US position in the backbone structure declined as a result. In contrast, India’s number of backbone edges grew significantly, from 9, in 2000, to 61, in 2021, eventually coming in first place, with nearly twice as many as second-place Thailand, making India the absolute core of the backbone network. China’s node degree in the backbone network showed a trend of first decline and then rise. In 2000, the number of China’s backbone edges was 20; in 2015, China’s out-degree of the original network was as high as 127, but the node degree in the backbone network was only 8; then, under the promotion of the “Belt and Road Initiative", China’s overseas rice trade shifted from a wide range of links to a large scale and high quality, and the number of backbone edges has gradually risen to reach 22 in 2021, ranking 6th globally, making it one of the Asian cores of the backbone network. In addition, there are some countries globally that are outside the rice-trade backbones, with fluctuating numbers of 148, 174, 170 and 160 nodes in the backbone structures in 2000, 2010, 2015 and 2021, respectively, and fewer countries in the South Pacific appearing in the backbone structures.

Overall, the backbone structures of the global rice-trade networks have expanded and evolved, showing a tendency to converge towards the core countries. In 2000, the backbone network was centered on Thailand and the United States, with backbone nodes and edges converging in Asia. Subsequently, the core nodes and edges of the backbone structure evolved with the changes in the rice-trade situation of various countries. By 2021, the backbone structure was formed with India as the core, Thailand and Pakistan as the secondary core, Italy, the United States, China and Vietnam as the key nodes, and the main nodes interconnected with each other and radiated to drive the backbone structure of the other regions, and the formation of their backbone networks in localized regions such as Asia and Europe.

## 4. Discussion and Implications

Economic globalization and trade liberalization have led to a significant expansion and intensification of the rice trade. The findings of this study reveal that certain key countries, namely India, Vietnam, Thailand, Pakistan, the United States, and China, exert substantial control over the global rice-trade networks—additionally, the production and export of rice display evident geographical concentration. Notably, the global rice-trade networks exhibit a polarization effect, making them susceptible to risk from sudden events such as natural disasters and political instability in these core countries. These risks can quickly propagate throughout the entire rice-trade network, thereby impacting the food-supply security of other nations [20]. Based on these results, governments and policymakers can implement measures in the following areas to ensure food-supply security and promote rice-trade cooperation.

Firstly, optimizing rice-trade cooperation and building a diversified rice supply system. (1) The existing trade cooperation pattern should be optimized, focusing on bilateral trade with core countries. As shown in Section 3.2, the global rice-trade networks are characterized by regional imbalance, and fluctuations in trade from core exporting countries can affect the rice supply security of other nations, especially those heavily reliant on rice imports. To mitigate trade frictions and the impact of large countries on rice supply dynamics, rice-importing and core exporting countries can establish long-term, stable trade relations through corporate foreign investment, intergovernmental and inter-enterprise project cooperation, and the signing of trade agreements. (2) The geographic proximity advantages should be fully utilized to enhance trade cooperation with neighboring countries. According to the analysis in Section 3.4, the global rice trade exhibits geographic proximity characteristics, leading to the establishment of regional rice-trade backbone networks in Asia and Europe. Meanwhile, Latin America is witnessing the emergence of intra-regional trade patterns. To strengthen the rice trade further, the relevant countries can consolidate and enhance trade relations with neighboring countries through regional trade agreements and cooperation platforms. Trade agreements can facilitate bilateral rice-trade cooperation among member countries by providing preferential tariff policies, relatively easy market access, and harmonized rules and standards. For instance, rice-importing countries in East and Southeast Asia, such as China and the Philippines, can capitalize on the preferential policies of the China–ASEAN Free Trade Area (CAFTA) and the Belt and Road Initiative (BRI) to prioritize establishing and maintaining stable trade and cooperative relations with neighboring rice-producing countries like Vietnam and Thailand. (3) A diversified rice supply system should be established and enhanced. In Section 3.3, through the calculation of Weighted Vertex Degree, we found that some West African countries have a large import scale of rice. For large rice-importing countries in West Africa and other regions, it is essential to establish a diversified rice supply system to mitigate potential supply risks arising from dependence on a single importing partner country. This involves expanding rice-importing channels by increasing the number of import-partner countries and distribution regions, thereby achieving diversification of trading partners and reducing vulnerability to supply disruptions caused by regional natural disasters and conflicts. At the same time, strengthening the domestic rice supply chain is crucial. Measures such as introducing overseas seeds, providing financial support, and expanding cultivation are needed to enhance rice self-sufficiency.

Secondly, promoting complementarity and coordination between rice and other grains. Alongside rice, wheat and maize are also vital food crops globally, contributing to nearly 50% of daily calorie intake [1]. These three grains are widely produced, consumed, and traded worldwide, complementing each other in meeting dietary needs. Prior research indicates that, like rice-trade networks, global wheat- and maize-trade networks exhibit significant disequilibrium and complexity, with a few countries exercising considerable control over trade. However, the core countries involved in the trade networks of different food crops vary. According to the previous analysis, India serves as the core country in the rice-trade network, while Pakistan, Thailand, Vietnam, China, the United States, and Italy play crucial roles. However, South and Southeast Asian countries like India, Vietnam, and Thailand do not stand out in the wheat- and maize-trade networks. Instead, the core exporting powers in the wheat-trade network include the United States, Russia, Canada, and Ukraine [40], while the maize-trade network consists of three trade blocs: the European trade bloc centered on France and other countries, the trade bloc centered on Brazil and Argentina, and the trade bloc centered on the United States [41]. The cultivation and trade of these three food crops are influenced by geographical proximity, making them susceptible to the impact of local natural disasters, regional conflicts, or pandemics. However, the core countries and key regions in the trade network differ for each food crop type, allowing the possibility of substituting and complementing food crops during regional emergencies. Consequently, countries can establish mechanisms for coordination and complementarity among multiple food crops, strengthening trade cooperation between major food-producing countries of different types. Additionally, large food-importing countries can develop a diversified food import system to mitigate the risk of disruption in the supply of a single food, which could adversely affect overall food security.

Thirdly, establishing a rice-trade monitoring and early-warning mechanism and improving the rice-emergency reserve system. As shown in Section 3.1, the global rice trade has been susceptible to various challenges, including extreme weather, the COVID-19 pandemic, price crises, and trade frictions. Additionally, regional conflicts have impacted global trade, increasing uncertainty in the rice market. Therefore, countries should take proactive measures. On the one hand, they should establish a global rice-trade monitoring and early-warning mechanism, which focuses on tracking the trade activities of major rice-trading-partner countries and dynamically monitoring price fluctuations and supply–demand relationships in the international rice trade. On the other hand, governments must strengthen the emergency rice stockpile and other essential food commodities. This step is crucial to prevent supply-chain disruptions in the face of unexpected events such as major public health incidents, regional conflicts, international tensions, and other emergencies that could lead to food crises and social instability.

Fourthly, improving multinational firms’ competitiveness and incorporation into the global production network. A few multinational enterprises control the global rice industry, and there is a mismatch between global rice production and major multinational enterprises. The headquarters of these multinational enterprises are concentrated in a few countries, such as the United States, India and The Netherlands, and in the world’s major rice-producing and consuming countries with rice production, processing, distribution and marketing departments that control the global rice flow. Rice-importing countries in regions such as West Africa can entice multinational enterprises to invest by providing incentive measures. For large rice-producing countries such as Thailand, China, Vietnam, and Indonesia, on the one hand, it is necessary to cultivate multinational enterprises with global competitiveness to form a global rice production, trade, and marketing network centered on their multinational enterprises; on the other hand, they must actively integrate into the global rice-flow network dominated by multinational enterprises in developed countries and utilize the spillover effect of foreign enterprises to upgrade the country’s rice production capacity.

## 5. Conclusions

Rice is one of the major food crops, and the rice trade plays a crucial role in regulating the global food supply and maintaining food security. Based on international rice trade data, this paper adopts various network analysis methods, including centrality, network density, global clustering coefficient, global efficiency and disparity filtering, to portray the temporal changes in the global rice-trade scale and the spatiotemporal evolution and network topologies of the global rice trade, and to comprehensively identify the changes in the status of different countries and trade relations within the global rice-trade networks. The main findings are as follows:

(1) The global rice-trade scale has experienced fluctuating growth over time. The trade volume initially exhibited steady growth, but during the food crisis in 2008, it experienced a significant leap. Since then, the trade volume has maintained a consistent trend, with both 2009 and onwards showing synchronized fluctuations in growth. The global rice-trade networks exhibit clear hierarchical features and obvious spatial imbalances. The trade networks have become increasingly complex, resulting in significantly improved network efficiency. This has formed a rice-trade pattern, with Asia serving as the primary source of exports and Africa as an important import market.
(2) Centrality indicators were used to quantify the positions of economies within the global rice-trade networks. According to the weighted degree centrality, Thailand, Vietnam, India, China, Pakistan, and the United States hold central positions in the global rice-trade networks, while the core of the African rice trade is experiencing dynamic changes. In-degree and out-degree analyses revealed that major rice-importing and exporting countries tend to have geographical concentration. European and North American countries, including Germany, France, the UK, the United States, Canada, The Netherlands, and Belgium, have a higher number of rice-importing partners. Meanwhile, the major rice-exporting countries are primarily in Asia, Europe, and North America, aligning with global rice production patterns.
(3) The global rice-trade networks have experienced significant expansion, leading to the evolution and enhancement of their backbone structures. The network backbones of the global rice trade are now taking shape, with major rice-exporting countries in Asia playing a central role, developed countries in Europe and North America occupying key positions, and rice-importing countries in Africa acting as complementary players. Among these, India constitutes the core of the backbone structure, while Thailand and Pakistan serve as secondary cores. Countries like Italy, the United States, China, and Vietnam also function as crucial nodes. The major nodes in the backbone structures are interconnected, radiating to other regions worldwide, and forming individual regional backbone networks within regions such as Asia and Europe.

Climate change has had significant impacts on global rice production and trade. From 2020 to 2023, La Niña occurred for three consecutive years, exacerbating droughts in the United States and Africa, increasing the likelihood of flooding in Southeast Asia [42], and potentially impacting global rice cultivation and trade. The study period of this paper is from 2000 to 2021, and the years 2022 and beyond have not been explored. Therefore, in future research, we will extend the period of the study to assess the impacts of climate extremes in recent years on the patterns and structures of the global rice trade and carry out research on the rice-trade system and food-security coping strategies under the influence of natural disasters, to enrich the academic community’s comprehensive knowledge of the global rice-trade system and supply-chain security. In addition, in future research, the study of trade flows of other food crops such as wheat and maize will also be developed.

## Figures and Tables

**Table 1:** Network characteristics of the global rice trade.

Network Description	2000	2005	2010	2015	2021
Graph size	1781	2233	2777	2854	2883
Network density	0.0767	0.0962	0.1196	0.1229	0.1242
Global clustering coefficient	0.3583	0.3665	0.3785	0.3923	0.4433
Global efficiency	0.0010	0.0009	0.0027	0.0039	0.0089

**Table 2:** Centrality indicators in the global rice-trade networks.

2000						2010					
Country	WDC	Country	IN_D	Country	OUT_D	Country	WDC	Country	IN_D	Country	OUT_D
THA	4.70	DEU	41	USA	116	THA	6.34	CAN	54	USA	152
USA	2.92	FRA	37	ITA	104	VNM	4.52	DEU	52	THA	143
CHN	2.36	CAN	37	THA	96	PAK	3.67	USA	50	CHN	136
VNM	2.01	RUS	36	CHN	90	USA	3.58	GBR	47	PAK	128
IDN	1.34	GBR	34	IND	87	IND	3.02	ZAF	43	IND	127
IND	1.34	USA	30	PAK	84	PHL	2.39	GHA	43	ITA	126
NGA	1.30	ZAF	30	JPN	69	ARE	2.08	FRA	42	VNM	112
IRN	1.15	ARE	29	GBR	66	BRA	1.65	DNK	40	JPN	89
ITA	1.06	ESP	29	ESP	64	SAU	1.30	ITA	39	FRA	86
PAK	1.04	CHE	27	FRA	60	IRN	1.13	IRL	39	ESP	83
**2015**						**2021**					
**Country**	**WDC**	**Country**	**IN_D**	**Country**	**OUT_D**	**Country**	**WDC**	**Country**	**IN_D**	**Country**	**OUT_D**
IND	10.03	NLD	60	THA	145	IND	12.23	NLD	73	IND	136
THA	8.05	GHA	56	USA	140	CHN	5.89	CAN	71	USA	130
VNM	4.93	FRA	55	IND	140	THA	4.41	FRA	66	THA	123
PAK	4.01	DEU	55	ITA	130	VNM	4.05	USA	59	CHN	117
CHN	3.76	GBR	53	CHN	127	PAK	2.97	GBR	59	ITA	116
USA	3.07	USA	52	PAK	127	USA	2.63	CHE	58	PAK	109
SAU	1.66	CHE	48	VNM	117	ETH	1.40	DEU	56	VNM	104
ARE	1.52	ZAF	44	ESP	94	BEN	1.40	BEL	54	ESP	90
SEN	1.17	DNK	44	GBR	90	NPL	1.37	ARE	54	JPN	80
CIV	1.14	BEL	42	FRA	89	KHM	1.31	ITA	49	FRA	78

## Data Availability

All data in this paper are available upon request.
